# Copper on chitosan-modified cellulose filter paper as an efficient dip catalyst for ATRP of MMA

**DOI:** 10.1038/s41598-021-87755-1

**Published:** 2021-04-15

**Authors:** Elham Feiz, Mojtaba Mahyari, Hamid Reza Ghaieni, Saeed Tavangar

**Affiliations:** Faculty of Chemistry and Chemical Engineering, Malek Ashtar University of Technology, Tehran, Iran

**Keywords:** Chemistry, Engineering, Materials science, Nanoscience and technology

## Abstract

Achieving an efficient catalyst in the ATRP system with a simple design, preparation from available materials, and high recyclability is a significant challenging issue. To attain the goal, herein, we used chitosan (CS)-modified cellulose filter paper (FP) as a green support for the synthesis of dip catalyst. The preparation of this catalyst involved surface treatment of the FP strips by CS coating through a dipping method, which increased the affinity of the substrate for adsorbing copper ions in the next step. The Cu@CS-FP catalyst was prepared without the requirement of any ligands. The synthesized dip-catalyst, in the form of the strips, was employed for the first time in the ATRP reaction of methyl methacrylate to assay catalytic activity. Catalytic insertion/ removal (ON/OFF) experiments were carried out during the polymerization. A reasonable control over the molecular weight with high conversion (68%) and polydispersity index of 1.32 under mild reaction conditions were obtained. Significantly, because of the facile separation of the catalyst, the amount of copper that remained in the polymer was very low (2.7 ppm). Also, the recyclability of the catalyst was investigated for five runs. The conversion in the final run was 64% without a loss of catalyst efficiency.

## Introduction

The controlled/ living radical polymerization (CRP) is the leading industrial method to prepare polymers. Currently, atom transfer radical polymerization (ATRP), as a transition metal-catalyzed CRP, is one of the effective procedures. The benefits of ATRP include wide applications in the preparation of well-defined polymers with sophisticated architecture, predicted molecular weight, and narrow polydispersity^1–4^. The ATRP is based on a redox process, catalyzed by a transition metal complex (especially copper) and a halide atom (usually Cl or Br)^5^. Transition metal catalysts create the radicals through reversible activation and deactivation of alkyl halide. The formation of radicals leads to subsequent monomer addition and the controlled polymer chain growth^6^. Reversible transfer between the growing radical chain and the dormant species is the foundation of the ATRP method^7^. The design of suitable catalysts, as the most important part of ATRP, to establish a dynamic equilibrium between propagating and dormant chains have attracted the attention of scientists^8^. In some recent reports, several catalysts consisting of copper-based catalysts with various ligands such as CuBr/PMDETA^9^, CuBr/Me_6_TREN^10^, CuBr/HMTETA^9^, etc. have been reported for ATRP reaction of MMA. Homogeneous catalysts due to well association with reactants is more efficient than heterogeneous catalysts^11^. On the other hand, several disadvantages such as recycling, separation, and reusing limit the application of homogeneous catalysts^12^. Typically, the catalyst/monomer molar ratio in the ATRP polymerization system is 0.001 to 0.01. This residual catalyst deeply colors the final polymers that needs purification after polymerization^13^. The notable disadvantages of ATRP are high-costs of post-polymerization purification for removal of the metal complex, the low catalytic activity, expensive ligands, and toxicity of final polymer^5,13^. Therefore, one of the most effective solutions for these problems is embedding the transition metal on suitable supports to improve stability, separation, and recycling^11,14^.

"Dip catalyst" is a nanocomposite thin film based on proper structural materials and noble metal nanoparticles^15^. Recently, dip catalyst by unique properties such as green and easy usage, excellent recovery, simple fabrication, and activation/deactivation reactions through insertion/deletion, has attracted much attention among other catalysts^11,16^. In 2016, Ikram and coworkers synthesized a dip catalyst by loading Ag nanoparticles on chitosan-coated filter paper support for the degradation reaction of nitroarene compounds. The fast separation of catalyst from the reaction media made the recovery process easy^17^. Dip-catalyst based on a gold nanoparticle–loaded filter paper composite showed high surface-enhanced Raman scattering (SERS) efficiencies and excellent recyclability for more than 20 continuous cycles^14^. The use of palladium nanoparticle-embedded polymer dip catalyst in the Suzuki − Miyaura reaction resulted in very high yield, TON and TOF, and the possibility of scale-up^11^. Dip catalyst, paper-based composite, afforded through dip-coating of FPs^17,18^. Dip catalysts as a new generation of catalysts in ATRP reactions with having extensive recyclability, not only cause to decrease toxicity and post-polymerization purification costs but also allow a controlled polymerization through the polymerization start/stop instantaneously by insertion/removal^15^.

Following the replacement of harmful chemicals with sustainable and environmentally friendly sources, the use of green catalysts has become a significant focus of interest for various research groups. The FP, the polymer with natural origin, due to properties such as stability, renewability, high availability, non-toxicity, surface functionality, and high surface area can be used as an active substrate for dip catalyst applications^18,19^. The FP with -OH functional groups is modified by two methods including chemical modification and physical modification^20,21^. Physical modification is preferred over chemical one due to the chemical method limitations such as time-consuming, specialized care, expertise, and advanced equipment for a successful modification^17,18,22–25^. Therefore, physically modified cellulose FP have found applications in a variety of fields including SERS sensor^22^, removal of hazardous pollutant ions from water^23^, wastewater treatment^18,24,25^, preparation of antibacterial papers^26^, determination of acetylsalicylic acid^27^, presentation of an optical sensor for ascorbic acid^28^, and dip catalyst for the degradation of nitroarene compounds^17^.

In this work, we followed three essential imperatives: (i) the catalyst should be safe, green, stable, easily synthesized from available raw materials and recyclable with a minimum level of polymer contamination; (ii) the selected chemicals should be having a high affinity to copper ions for an acceptable chelating; (iii) developing an efficient and environmentally friendly ATRP reaction, with good conversion, and not using conventional high-cost catalysts.

According to the reviews and studies reported in the literature about ATRP reactions, one of the most critical limiting issues for the applicability of ATRP is the toxicity of the residual copper in the final product^29–33^. So, the design and preparation of an efficient catalyst as the ATRP key can play a vital role in developing ATRP for biological goals^8,34–36^. In recent years, biological macromolecules (especially cellulose) have attracted increasing attention as catalyst supports due to following reasons: (i) biopolymer-supported catalysts are inexhaustible, chemically stable, inexpensive, biocompatible, and biodegradable; (ii) the support with hydroxyl groups and high functionalization forms an excellent polymeric skeleton for the stabilization of copper ions as well as other transition metals; (iii) they have excellent characteristics such as hydrophobicity, large chemical modifying capacity, and high surface area^37–39^. So, FP can be applied as green support in the preparation of dip catalyst^18,40^.

The green, efficient, stable, economical, and reusable catalysts were modified for ATRP reactions. We introduced CS to FP as available and green support with the high surface area, recyclable for copper (I) stabilization^17^. The prepared Cu@CS-FP as a dip catalyst was examined for ATRP reaction of methyl methacrylate as model polymerization reaction which represented the high catalytic activity and recyclability. It should be noted that this is an example of the use of dip catalyst as a novel catalyst for ATRP reactions. The clean and well-defined polymers with low molecular weight distribution were obtained, without the need for purification.

## Experimental

### Materials

FPs were purchased from Whatman Co. The diameter and thickness of FPs were 12.5 cm and 0.3 mm, respectively. They were cut into rectangular strips with dimensions of 0.5 × 3.5 cm^2^. Methyl methacrylate (MMA, 98%; Merk) was distilled over CaH_2_ under reduced pressure to remove the inhibitor and stored at − 15 °C before utilization. CS was purchased from Sigma-Aldrich Co. Copper (I) bromide (CuBr, 99%), Ethyl α-bromoisobutyrate (EBiB, 98%), and Acetic acid (AcOH, 99%) were obtained from Merck Co.

### Measurements

Fourier transform infrared (FT-IR) spectra of the samples were performed on attenuated total reflectance Perkin Elmer (spectrum100) ATR-FTIR spectrometer in the wavenumber range from 400 to 4000 cm^-1^. Data were baseline-corrected and smoothed in the OPUS software. X-ray diffraction (XRD) patterns to studying crystal structures of samples were analyzed using an XD-3A diffractometer (Philips, The Netherlands) with a Cu Kα radiations (λ = 0.154 nm) source on the 2θ range from 10° to 80° at room temperature. To determine morphology and surface imaging, SEM analysis was carried out by JEOL JSM7600F of Japan equipped with a backscattered electron, secondary electron detector, and an EDAX energy-dispersive X-ray spectrometer. The elemental distribution and weight percent were obtained from EDX line-scanning of the chemically modified cell wall. Thermogravimetric analysis (TGA) was carried out using STA 1500 instrument at a heating rate of 10 °C min^−1^ under air for studying of thermal decomposition of dip catalyst. Varian 3900 gas chromatograph (GC) (Varian Iberica, Madrid, Spain) was used to determination of the monomer conversions by evaluation of the concentration of the remaining monomer using n-decane as an internal standard. GC was formed from the split/splitless capillary injection port and flame ionization detector (FID). A CPSil-8 fused silica capillary column (25 m × 0.32 mm i.d. and 0.52 mm film thickness) was utilized from Chrompack. Molecular weights and polydispersity of polymers were measured on a GPC Agilent 1100 model equipped with an Agilent Waters 1515 Isocratic HPLC pump, a Waters 2414 refractive index detector, and PLgel 3 µm 300 × 7.5 mm column. The measurements were performed at 35 °C using LiBr-added DMF ([LiBr] = 15 mM) as eluent (flow rate: 1.0 mL/min). Calibration of the system was done by linear poly (methyl methacrylate) standards.

### Preparation of modified filter paper (CS-FP)

The surface modification of FP was performed physically by CS. First, the CS aqueous solution with a concentration of 1 wt% was prepared simply by dissolving flaked CS in a 2% v/v acetic acid solution^17,18^. In the next step, FPs were cut into rectangular strips with 0.5 × 3.5 cm^2^ dimensions and immersed into CS solution for 3 h^17^. After this time, to avoid the blocking of the pores, the excess solution was removed from the over-soaking strips, and they took out. Finally, they dried at 60 °C overnight for the next-step use.

### Preparation of Cu@CS-FP strip catalyst

0.02 g CuBr in 5 mL of dry acetonitrile solvent (5.0 ml) was added to a Schlenk flask and CS-FP strips were transferred to it. Then, the flask was sealed with a rubber septum, degassed and backfilled with nitrogen three times, and refluxed for 23 h under nitrogen. After that, the strips were washed out with dry acetonitrile to remove the loosely bound and un-reacted Cu ions and dried at 60 °C for 14 h in the oven.

**Figure 1:**
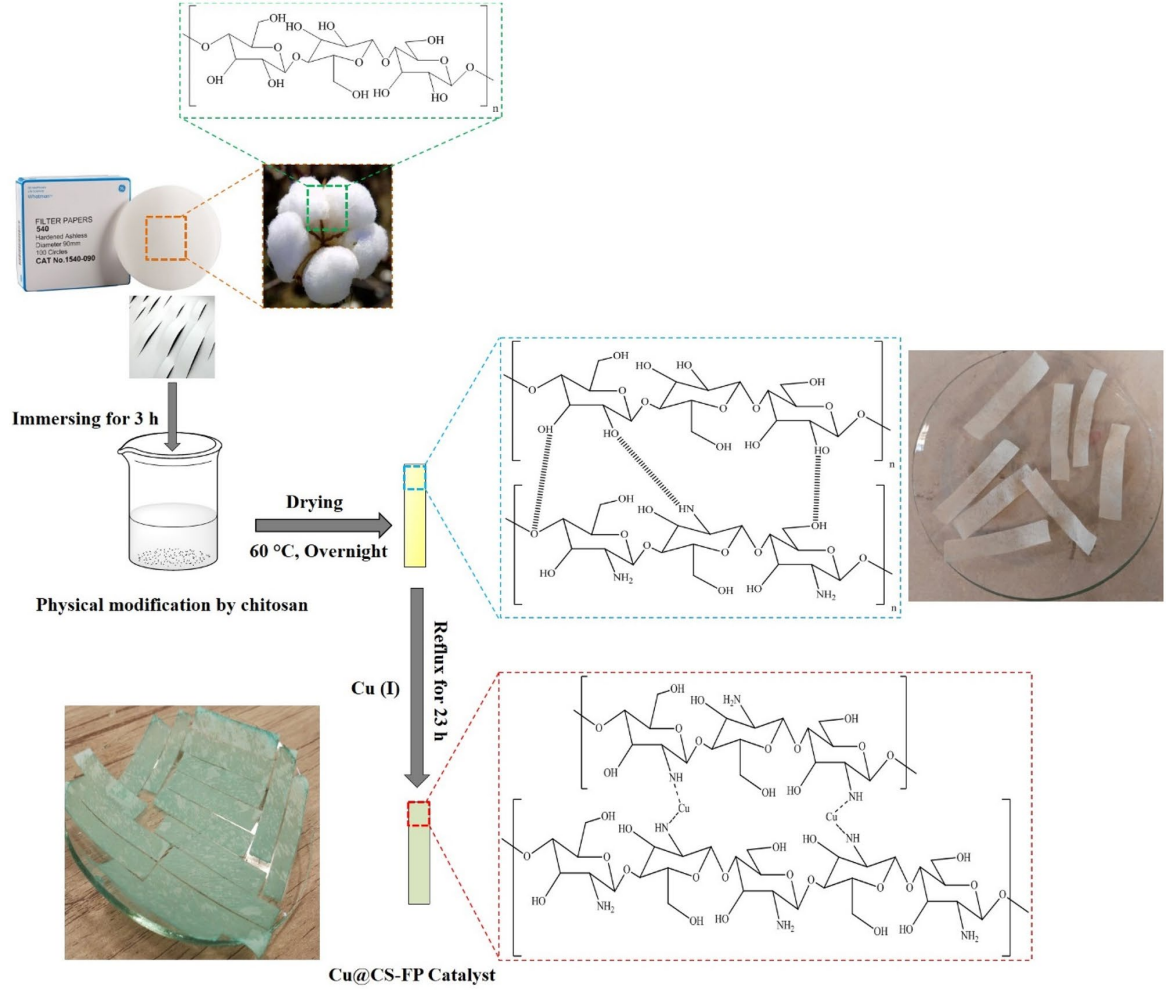
The preparation steps of Cu@CS-FP dip catalyst.

Figure 1 illustrates the preparation process of the Cu@CS-FP dip-catalyst, including two steps. First, FP strips was dipped in 1 wt% CS aqueous solution for 3 h according to the literature by Kamal et al. ^18^. The FP strips were modified physically by CS by forming a thin coating film upon the FP via the dipping method. The electrostatic interaction between the negative and positive charges of cellulose and CS binds them together^17^. By the coating process, the physical modification was done to raise the affinity of support for the chelating of ions. According to previous studies, CS has the –OH and –NH_2_ functional groups, which results in a good affinity for various metallic ions. Subsequently, due to the high affinity of CS to copper ion^41^, Cu(I) was chelated to the CS by the coordination bond^42–46^. Based on the previous reports, there are two possibilities for the coordination bond between copper and chitosan, including a bridge and pendant models^41^. The bridge model was attributed to the Cu binding to various nitrogens from within the same chain or from adjacent chains. The pendant model describes a one-to-one pendant-like bond of copper to an amino group. Gritsch et al. recently found some evidence for the bridge model^46^. To investigate the Cu ratio in the as-synthesized CS-FP, AAS was employed which showed 24.2 wt% Cu loading. Various analyses have been conducted to confirm the successful preparation and to study the structure and composition of the samples.

### General procedure for the ATRP reaction of methyl methacrylate

**Figure 2:**
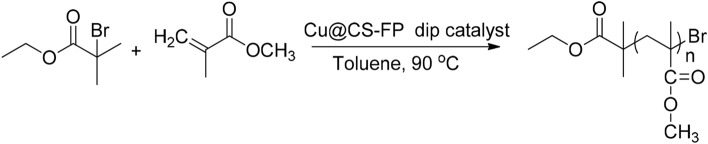
ATRP reaction of methyl methacrylate.

In a typical run, toluene (3.0 mL) was deoxygenated and introduced to a Schlenk flask fitted with a magnetic stirring bar. Then, MMA (2.13 mL, 18.6 mmol), EBiB (13 μL, 0.093 mmol), and Cu@CS-FP (20 mg) were transferred to it. Degassing of the reaction mixture before sealing in a vacuum was done by three freeze–pump–thaw cycles and subsequent bubbling dry N2 for 30 min. The mixture was placed in an oil bath and stirred at 90 °C. After the reaction time, the Cu@CS-FP strip catalyst was extracted simply from the polymer. Afterward, the polymer was regained by sedimentation in cold methanol and drying under a vacuum (Fig. 2).

## Results and discussion

### Characterization of Cu@CS-FP catalyst

**Figure 3:**
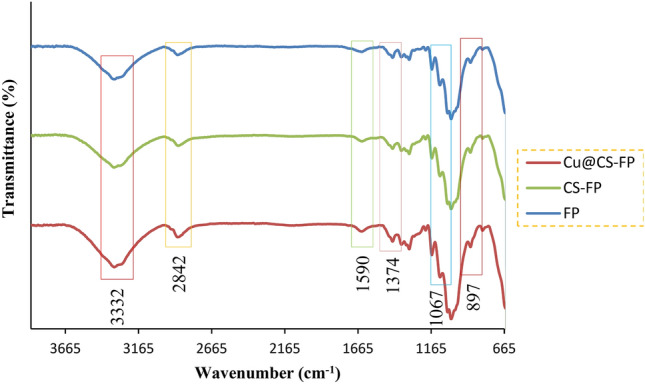
FT-IR spectra for FP, CS-FP, and Cu@CS-FP.

FT-IR spectra of FP, CS-FP, and Cu@CS-FP were shown in Fig. 3, respectively. As can be seen, the FTIR spectra of all samples are roughly similar. It showed peaks at 3332 cm^-1^ (O–H stretching)^47^, 2842 cm^-1^)C–H stretching), 1590 cm^-1^ (N–H vibration)^48^, 1374 cm^-1^ (C-H and C-O vibrations in the polysaccharide rings of cellulose)^49^, 1067 cm^-1^ (C–O–C pyranose ring skeletal vibration), and 897 cm^-1^ (β-glycosidic linkages)^50,51^. Because of low CS coating and Cu ions in the samples, the interaction among them was weak and not detected by the FT-IR technique.

**Figure 4:**
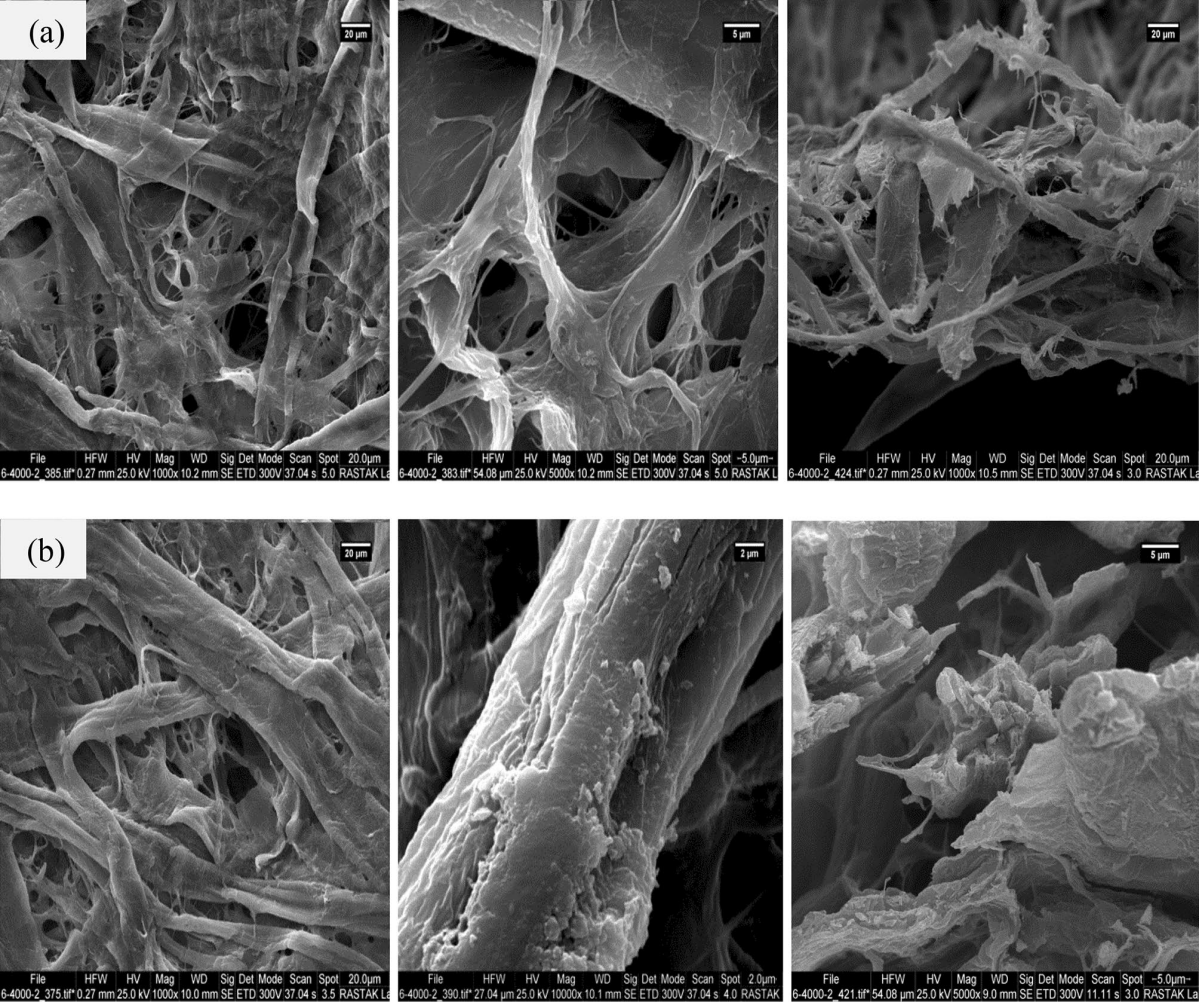
FE-SEM images of the **(a)** CS-FP, and **(b)** Cu@CS-FP. The images from left to right in order: low magnification, high magnification, and cross-sectional.

The morphology and characterization of the surface for CS-FP and Cu@CS-FP were investigated using SEM (Fig. 4a,b). FE-SEM images of the samples in order from left to right, include low magnification, high magnification, and cross-sectional, respectively. Microfibers along-with nanofibrils of FP can be seen in all of the images. The FE-SEM image for CS-FP is similar to the untreated FP because the use of dilute CS solution makes the porosity unchanged^18,52,53^. The CS formed a smooth, thin film on the cellulose surface of FP by fine penetrability. The electrostatic interaction among CS with the positive charge and FP with the negative charge bounded them together^52–54^. Figure 4b shows the FE-SEM image of Cu@CS-FP. As shown in surface imaging, the Cu@CS-FP in comparison to the CS-FP indicated high homogeneity and less porosity. Also, based on the cross-section SEM image of Cu@CS-FP, a smooth texture was observed and thickness remained unchanged.

**Figure 5:**
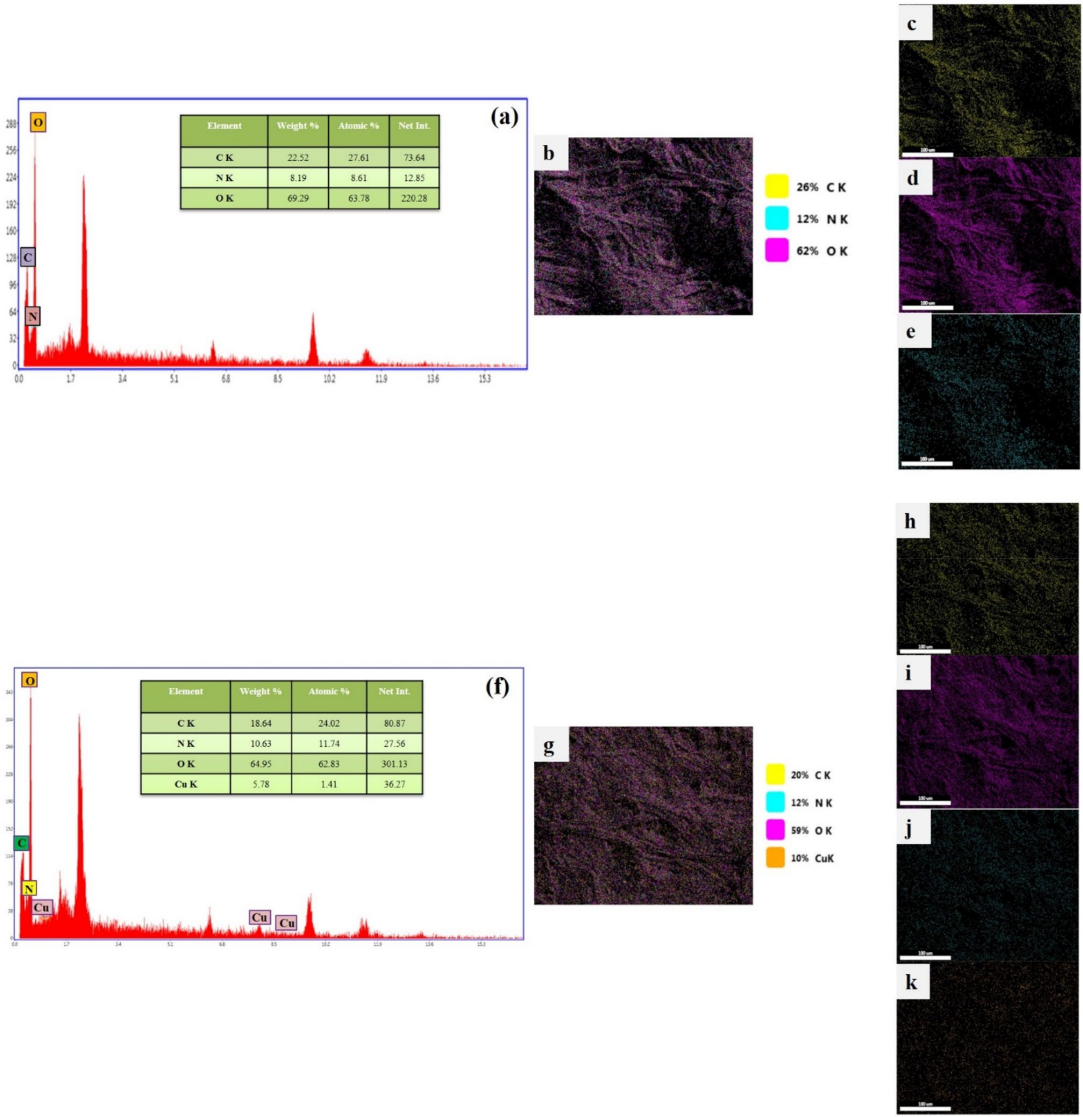
**(a)** The EDX spectrum of CS-FP; **(b)** overall mapping elements: corresponding to carbon **(c)**, oxygen **(d)**, and nitrogen **(e)**. **(f)** The EDX spectrum of Cu@CS-FP; **(g)** overall mapping elements: corresponding to carbon **(h)**, oxygen **(i)**, nitrogen **(j)**, and copper **(k)**.

We studied samples through elemental analysis and Energy Dispersive X-ray analysis (EDX) mapping analysis to further confirm the successful formation of dip catalyst, and distribution of copper and CS onto the FP cellulose microfibers. Elemental analysis displayed the weight percent of carbon, nitrogen, oxygen elements in the CS-FP sample, and those of the Cu@CS-FP sample as well as the weight percent of the copper element (Fig. 5). The carbon, nitrogen, and oxygen signals were appeared due to the organic nature of the FP and CS. Besides, according to EDX analysis, copper is present in the Cu@CS-FP sample without considerable contaminations from other elements especially sodium or chlorine^55^. The uniform distribution of copper in the Cu@CS-FP showed that it has stabilized by CS successfully. Figure 5 has indicated that nitrogen and copper were present at the surface of FP.

**Figure 6:**
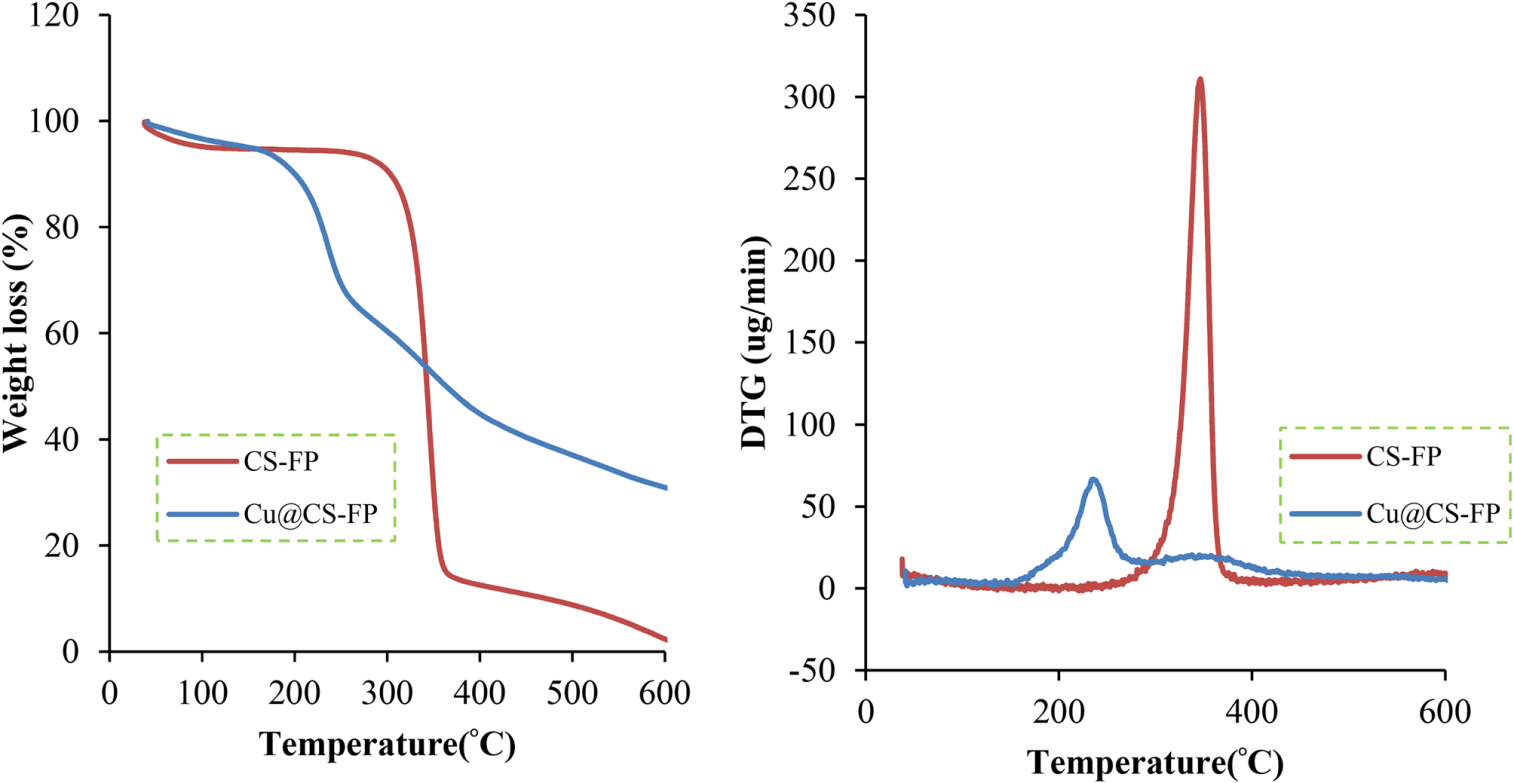
TGA and DTGA curves of the CS-FP, and Cu@CS-FP.

Figure 6 shows the TGA and DTA thermograms of the CS-FP and Cu@CS-FP. First, by increasing the temperature to 100 °C, CS-FP showed 3% weight loss due to moisture elimination. This sample is stable up to 300 °C which indicated no more weight loss in this range. The polymer decomposition was accomplished after 300 °C which led to 91% weight loss. The weight loss in temperature below 180 °C in the TGA curve of Cu@CS-FP was attributed to the loss of absorbed water. Because of the interaction of metal ions with amino groups in the Cu@CS-FP sample and subsequently loss in hydrogen bonds, the Cu@CS-FP showed a higher decomposition rate after Cu loading^56^. Moreover, the inherent nature of Cu caused the low activation energy in the depolymerization reaction^57–59^. The decomposition was occurred after 180 °C causing the 62.2% weight loss. The difference between the whole weight losses of CS-FP and Cu@CS-FP was about 28.8% which can be attributed to the Cu loading.

**Figure 7:**
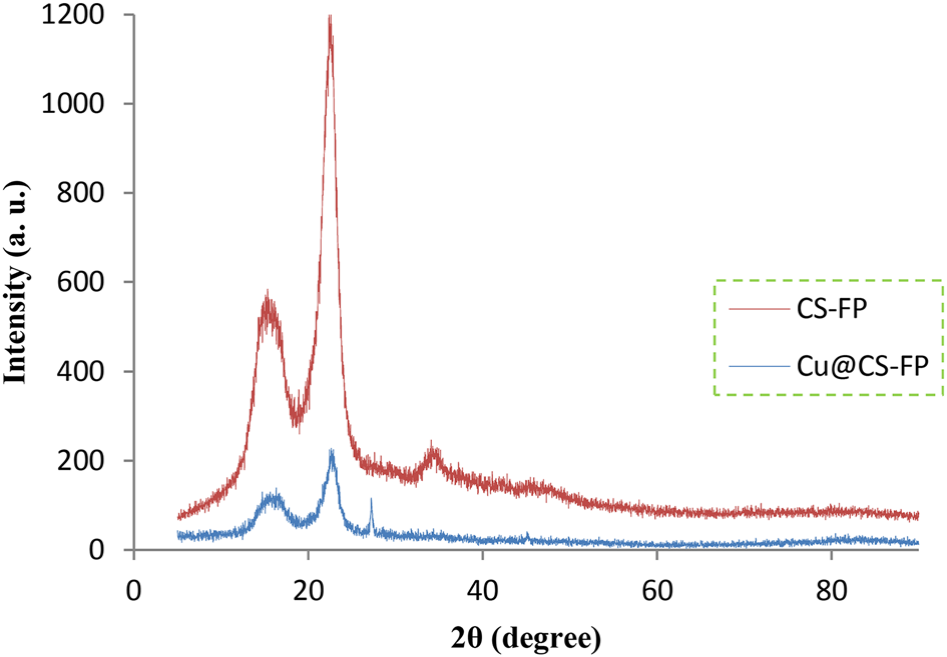
XRD patterns of the CS-FP, and Cu@CS-FP.

In Fig. 7, the XRD patterns of the CS-FP, and Cu@CS-FP are shown. The CS-FP pattern indicated peaks at 2θ value of 14.8°, 16.5°, and 22.3° corresponding to the (1–10), (110), and (200) of the cellulose-I crystal structure (JCPDS. No. 03-0226)^17^. According to the literature, the peak at 34.1°, which appeared randomly, can be a composite of several reflections and (004) is not the dominant contributor^60^. By comparison of CS-FP, Cu@CS-FP, and cellulose patterns, CS may be coated in the form of an amorphous layer on the FP surface^17,18^. The physical modification does without the changes in the crystal structure of cellulose. The XRD pattern of the Cu@CS-FP was showed a Bragg reflection at 2θ = 27.1°, which was absent in the XRD pattern of the CS-FP. This peak is indexed as (111) and probably related to the face-centered cubic structure of Cu. Due to the low content of the Cu in the Cu@CS-FP strip, the rest of the peaks of Cu were not observable in the pattern.

### The catalytic activity of Cu@CS-FP dip catalyst for ATRP reaction

To investigation the efficiency of Cu@CS-FP, the ATRP reaction of methyl methacrylate in the presence of ethyl α -bromoisobutyrate (EBiB) initiator and toluene solvent as a model reaction. The reaction was performed in the presence of various amounts of the catalyst (Table 1). For a better comparison, the molar ratio of monomer to initiator was considered similar in all experiments. When the experiments were carried out in the absence of dip catalyst, the reactions failed to produce any polymer even after 48 h (Table 1, Run 1). Hence, the presence of Cu@CS-FP dip catalyst in the ATRP of MMA is essential, and polymerization drove by it. With increasing the amount of catalyst at 24 h reaction time, the monomer conversion increased significantly from 9 to 68%, and polydispersity tended to narrow from 1.51 to 1.32 (Table 1, Run 2–4). A further increase in the amount of catalyst had the opposite effect, and only 19% conversion was achieved even after 30 h of reaction (Table 1, Run5). The experiments with the optimal amount of catalysts were repeated for longer times. The results showed that increasing the duration leads to low conversion and high molecular weight distribution (Table 1, Run6–7).

**Table 1:** ATRP reaction using Cu@CS-FP as a dip catalyst.

Entry^a^	[M]_0_/[I]_0_^b^	Amount of catalyst	Reaction time (h)	Conv.^c^ (%)	*M*_n,th_^d^ (g mol^−1^)	M_n,GPC_^e^ (g mol^−1^)	M_w_/M_n_^f^
1	200/1	–	48	–	–	–	–
2	200/1	0.008	24	9	1689	1752	1.51
3	200/1	0.01	24	16	2350	2466	1.46
4	200/1	0.02	24	68	14,100	14,800	1.32
5	200/1	0.03	30	19	5743	3980	1.78
6	200/1	0.02	32	49	9850	8570	1.54
7	200/1	0.02	38	30	6130	4790	1.63

^a^Polymerization conditions: MMA was used as a monomer, and EBiB was used as the initiator, dip catalyst, Monomer/solvent = 1/1 (v/v), N_2_ atmosphere, T = 90 °C.

^b^[M]: Monomer, [I]: Initiator.

^c^Monomer conversion measured by gas chromatography.

^d^Theoretical number average molecular weight (M_n_), computed via monomer conversion. M_theo_ = M_initiator_ + α[M]_0_/[I]_0_; α demonstrates monomer conversion.

^e^M_n_ determined by gel permeation chromatography.

^f^Molecular weight distribution.

**Figure 8:**
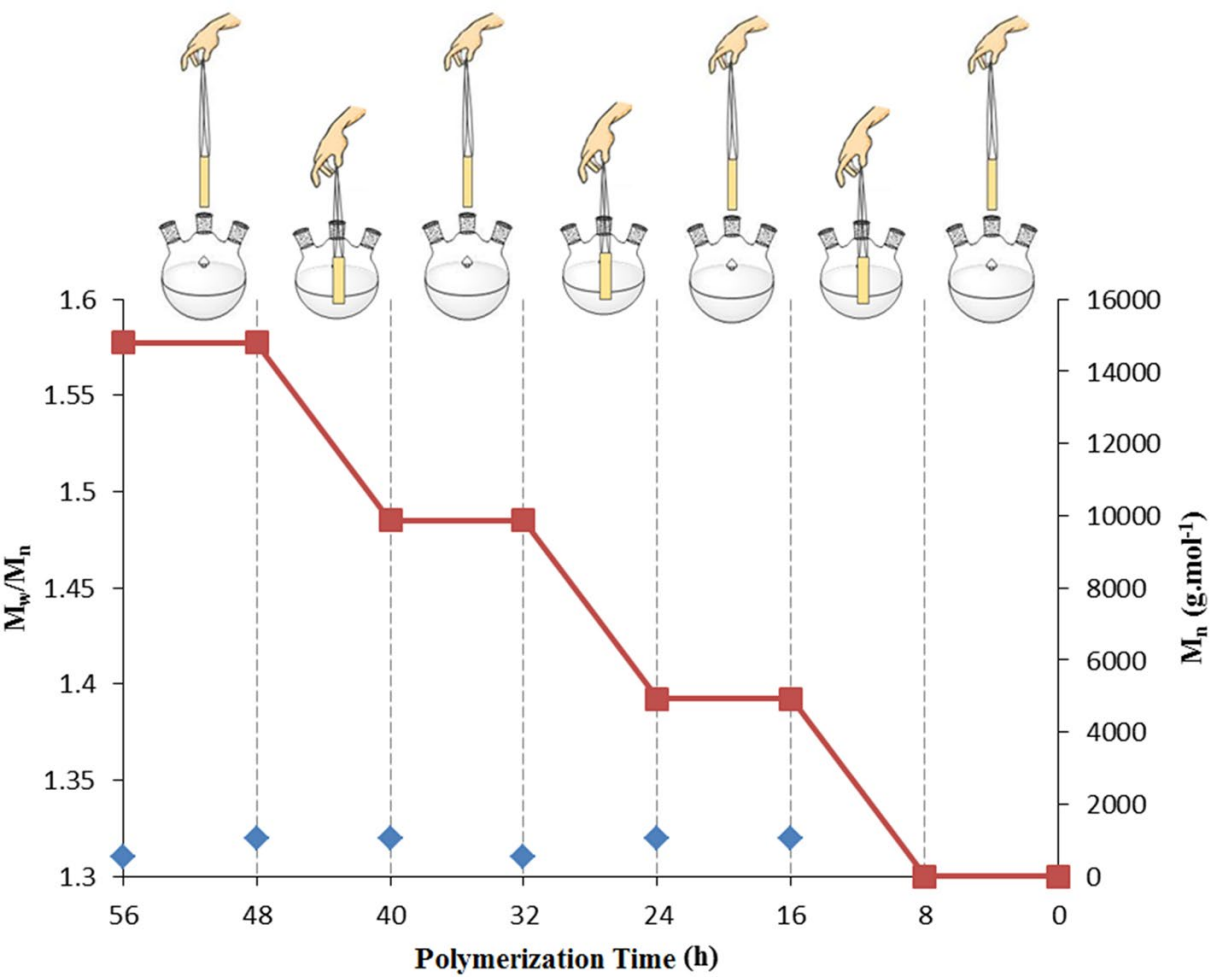
The role of catalyst in polymerization using the Cu@CS-FP catalyst.

The effect of the catalyst on the polymerization was evaluated by the insertion and removal of the catalyst (Fig. 8). Catalyst insertion/ remove (ON/OFF) experiments were performed during the polymerization alternatively for every 8 h. When the dip catalyst is removed, no polymer was observed and stopped the reaction due to a low concentration of the active radical species. By each insertion, the reaction was restarted and controlled polymerization was observed with a molecular weight (Mn) of 4933 g mol^−1^ that was analyzed by GPC. Good temporal control over the polymerization was observed by repeating these cycles. Significantly, the as-obtained polymers had a low polydispersity index (1.32).

### Residual copper

As mentioned earlier, one of the most drawbacks of the ATPR is residual copper in the polymer. Therefore, to determine the amount of copper in the final product, atomic absorption spectroscopy (AAS) was applied. After the reaction time elapsed, taken out of the catalyst strips, and polymer sedimentation from methanol, the analysis revealed that about 2.7 ppm of copper remained in the final product.

The comparison of the prepared catalyst for the ATRP reaction of methyl methacrylate with previously reported copper-based catalysts, respect to the initiator, solvent, reaction time, conversion percentage, and molecular weight distribution, have been provided (Table 2). ATRP of MMA using 3-bromo-3-methyl-butanone-2 (MBB) as an initiator in the presence of CuBr and different ligands as a catalyst in toluene solvent at 90 °C was presented according to previous researches (Table 2, Run 2–7). Despite homogeneous catalysts, the rate of polymerization was relatively slow in the case of bidentate ligands (Table 2, Run 2–3). The tridentate N-donor ligands caused better results (Table 2, Run 4–5). The reaction was very fast and uncontrolled with multidentate linear amines (Table 2, Run 6). The copper (I) with 2,6-bis(4,4-dimethyl-2-oxazolin-2-yl)pyridine (dmPYBOX) was formed a more stable complex but disturbed the equilibrium dynamics of the reaction which resulted in low molecular weight (Table 2, Run 7). Also, the polymerization of MMA catalyzed with Cu complex, and EBiB as initiator only obtained a rather low conversion (6.1% in 10 h, and 16.7% in 6 h) (Table 2, Run 8–9).

**Table 2:** Comparison of the results obtained from Cu@CS-FP with various types of copper-based catalysts for the ATRP reaction of methyl methacrylate.

Entry	Catalyst	Initiator	Solvent	Time (h)	Conv. (%)	PDI	Ref
1	Cu@CS-FP	EBiB	Toluene	24	68	1.32	This work
2^a^	[CuBr]/[NPPI^b^]	MBB^c^	Toluene	5.5	3	1.06	^9^
3^a^	[CuBr]/[dnNbpy^d^]	MBB	Toluene	5.5	1	1.07	^9^
4^a^	[CuBr]/[PMDETA^e^]	MBB	Toluene	5.5	98	1.34	^9^
5^a^	[CuBr]/[BPIEP^f^]	MBB	Toluene	5.5	73	1.26	^9^
6^a^	[CuBr]/[HMTETA^g^]	MBB	Toluene	5.5	98	1.41	^9^
7^a^	[CuBr]/[dmPYBOX^h^]	MBB	Toluene	5.5	20	1.36	^9^
8^i^	[CuBr]/[Me_6_TREN^j^]	EBiB	–	6	16.7	2.83	^10^
9^i^	[CuBr]/[TPEN^k^]	EBiB	–	10	6.1	–	^10^

^a^Polymerization conditions: 90 °C.

^b^NPPI: *N*-(*n*-propyl)-2-pyridylmethanimine.

^c^MBB: 3-Bromo-3-methyl-butanone-2.

^d^dnNbpy: 4,4^′^-Di (*n*-nonyl) 2,2′-bipyridine.

^e^PMDETA: *N,N,N′,N′,N′*′-pentamethyldiethylenetriamine.

^f^BPIEP: 2,6-Bis[1-(2,6-diisopropylphenyliminodiisopropylphenylimino)ethyl]pyridine.

^g^HMTETA: (*N,N, N,N,N,N*-hexa methyltriethylenetetramine).

^h^dmPYBOX: 2,6-Bis(4,4-dimethyl-2-oxazolin-2-yl)pyridine.

^i^Polymerization conditions: 70 °C.

^j^Me_6_TREN: Tris[2-(dimethylamino) ethylamine.

^k^*N,N,N′,N*′-tetrakis(2-pyridylmethyl) ethylenediamine.

### Recycling Cu@CS-FP

**Figure 9:**
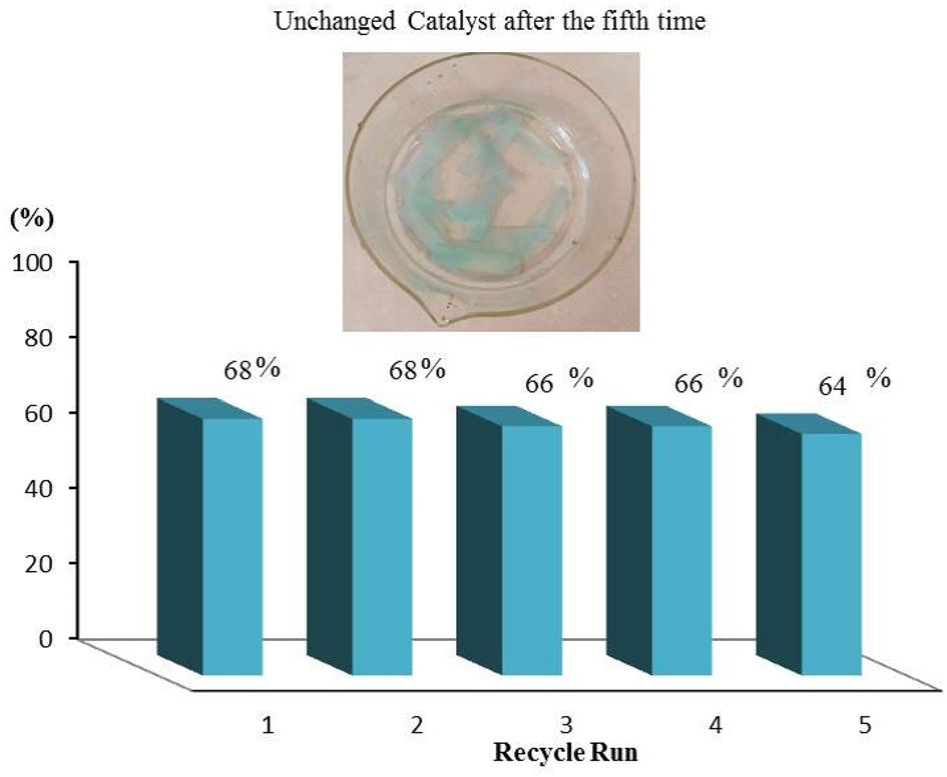
The outcome of recycling on the catalytic efficiency of the Cu@CS-FP dip catalyst.

Catalysis is a kinetic process. To determination the recoverability, deactivation, and stability of the catalyst, kinetic data must be appraised but traditionally recognized by the reaction yield or conversion after a long time in every run. Checking long-term data acquisition is not a reasonable way ^61^. We investigated the recyclability of the prepared catalyst and the leaching of the active sites in the reaction media via several tests. The ATRP reaction of the methyl methacrylate monomer was studied for five cycles, and the conversion percent calculated to any run after 7 h. Figure 9 shows that the catalyst is highly stable, in which the conversions after every run did not change considerably.

## Conclusions

We successfully prepared a recoverable dip catalyst based on CS-FP for ATRP reaction. Strong affinity between the copper ions and CS were caused to an acceptable and stable chelating. ATRP of MMA in presence of Cu@CS-FP using EBiB as initiator under a mild condition resulted in polymers with high conversion (68%) and relatively narrow molecular weight distributions (M_w_/M_n_ ≤ 1.32). Significantly, the growth of polymer chains can be switched to start/stop by insertion/removal of dip catalyst as an external stimulus. In each insertion of dip catalyst, the monomer conversion was ≈17% with M_n_ of 4933 g mol^−1^ analyzed by GPC. The recyclability of dip catalyst was investigated by five runs. The conversion in the fifth run was measured 64% without significant loss of activity. Also, the residual copper in polymers was determined to be approximately 2.7 ppm, which is a real improvement in the ATRP reactions. Due to the simplicity, environmentally friendly, and recoverability of the catalyst, it can be utilized in other ATRP reactions.
